# Dual-Energy X-Ray Absorptiometry Investigation of Fat Mass and Lean Mass in 193 Male Elite Youth Soccer Players

**DOI:** 10.3390/jfmk11040417

**Published:** 2026-10-13

**Authors:** Valentina Cavedon, Carlo Zancanaro, Chiara Milanese

**Affiliations:** Laboratory of Anthropometry and Body Composition, Department of Neurosciences, Biomedicine and Movement Sciences, University of Verona, 37134 Verona, Italy; carlo.zancanaro@univr.it (C.Z.); chiara.milanese@univr.it (C.M.)

**Keywords:** football, percentile, body composition, maturity offset

## Abstract

**Background:** Youth soccer is practiced worldwide. However, body composition during youth soccer practice has received limited attention despite its well-known impact on performance. **Methods:** Dual-energy X-ray absorptiometry (DXA) estimated whole-body and regional fat and fat-free soft tissue mass, and percent fat mass in a large sample (n = 193; 172 White, 21 Black) of male elite youth soccer players aged 14–19 years competing in an Italian Serie A Club. The role of playing position in shaping body composition was also explored. **Results:** Black players had higher appendicular fat-free mass index (*p* = 0.009) and lower total body less head (TBLH) fat mass index (*p* = 0.050) than White players. Within the entire sample of players, analysis of covariance (ANCOVA) conducted using several confounding variables as covariates showed a statistically significant effect of playing position for TBLH fat-free soft tissue mass (*p* = 0.049) and appendicular fat mass (*p* = 0.039), with the goalkeepers showing higher appendicular fat mass than defenders (*p* = 0.031). Also, playing position showed a significant effect on TBLH fat-free mass index (*p* = 0.044). Effect sizes for statistically significant ethnic differences ranged from η^2^_p_ = 0.02–0.06, whereas positional effects ranged from η^2^_p_ = 0.05–0.21, indicating that several, but not all, observed effects were small in magnitude. Descriptive percentiles for demographic and body composition variables and indices were calculated in three two-year age classes for White players only, because of the limited number of Black players. **Conclusions:** In our cross-sectional, single-club sample of male elite youth soccer players, DXA analysis provided descriptive data on fat- and lean-mass variables overall and according to playing position. Findings related to ethnicity should be interpreted cautiously because of the limited number of Black players. The percentiles provided for White players may serve as descriptive benchmarks for youth players.

## 1. Introduction

Youth soccer has received increasing attention over the past several years. A PubMed search over the last 25 years yielded 8250 published papers dealing with youth soccer, with a substantial increase year by year from the 54 papers published in 2000 and the 856 in 2024, indicating a growing interest in this topic. In the same time interval, 16,044 papers were found in the same database when searching for the search term “soccer” alone. Accordingly, papers on youth soccer investigations accounted for about 51% of the total. Surprisingly, when the search term “body composition” was added to the search string, only 367 papers out of 8250 (about 4% of the total) were found in the database, despite body composition having long been recognized as a critical factor in performance for both adult [1] and youth [2] soccer players. In a recent meta-analysis [3], lower percent fat mass (%FM) has been shown to discriminate higher-level players at different competitive levels. In youth soccer players, %FM was negatively associated with field-based performance tests [4]. Accordingly, information on the body composition characteristics of elite soccer players is relevant for helping players reach optimal performance potential [5] and monitoring the effects of conditioning or training.

Dual-energy X-ray absorptiometry (DXA) is the reference technique for body composition evaluation in athletes [6], yielding a three-compartment model (fat mass [FM], fat-free soft tissue mass [FFSTM], and bone mineral). DXA is increasingly used to evaluate and monitor body composition in soccer players [7,8] and was used in this work. Compared with commonly used field methods such as skinfolds and bioelectrical impedance analysis, DXA allows the assessment of whole-body and regional body composition, including FM, FFSTM, and bone mineral density [6]. Because body composition estimates may differ according to the assessment method used [8], DXA-derived data may provide useful method-specific descriptive benchmarks, particularly when regional tissue distribution is of interest, although their practical use is limited to settings where DXA is available. Previous DXA studies have also provided relevant information on body composition in youth soccer players, although they have addressed different age groups and study objectives. Milsom et al. [7] compared whole-body and regional body composition among first-team, U21, and U18 players, whereas Hannon et al. [9] examined body composition across academy age groups, with particular emphasis on growth, maturation, and resting metabolic rate. Portella et al. [10] provided DXA-derived reference values according to maturity status in 206 Brazilian players aged 11–16 years. More recently, Łuszczki et al. [11] examined body composition according to playing position in 189 youth soccer players aged 9–19 years.

These studies provide important information, but differ in age range, study purpose, and the body composition variables considered. Data combining whole-body and regional FM and FFSTM, stature-adjusted indices, positional comparisons, and age-stratified distributions in players during early-to-late adolescence remain limited.

Ethnicity has long been known as a factor affecting body composition in the healthy general population [12,13] and the sporting population [14]. At the same time, current guidance emphasizes that such categories are heterogeneous and may reflect social, environmental, and demographic factors in addition to other sources of individual variability. Moreover, the increasing mobility of young elite soccer players across championships prompts attention to ethnicity. Therefore, ethnic differences in soccer players should be considered when investigating their body composition. Further, body composition may be affected by a player’s playing position [15], and identifying positional differences in body composition would be beneficial for shaping training and nutritional interventions aimed at optimizing performance.

The aim of this cross-sectional study was therefore to explore the hypothesis that whole-body and regional FM and FFSTM in male youth soccer players aged 14–19 years may be affected by playing position and ethnicity. A further aim was to provide descriptive percentile values for body composition variables in players.

## 2. Materials and Methods

### 2.1. Study Sample

We collected a convenience sample of male elite soccer players aged 14 y–19 y from the youth squads of a Club competing in the Italian Serie A for the scope of multiple investigations. The study was conducted in accordance with the Helsinki Declaration and approved by the local ethics committee (2019-UNIVRCLR-0422326). Informed, signed consent was obtained from all participants and from at least one parent for minors. The sample size for one-way analysis of covariance (ANCOVA) with a maximum of three covariates (see below) was calculated a priori with G*Power software (version 3.1) [16]. Setting a medium effect size (Cohen’s f) of 0.25 with alpha = 0.05 and power = 0.80 with four groups (goalkeeper, defender, midfielder, forward), the calculated total sample size was n = 179. Where needed, participants were analyzed according to the four basic playing positions quoted above. Given the aims of this work, further fractionation of the study sample into sub-positions, such as central and lateral defenders, was deemed unnecessary. Players trained 4/5 times a week for 2 h (<18 y) or 5/6 times a week for 2 h (<20 y) and had a championship match one time a week during the season.

### 2.2. Procedures

All examinations were held in the summer before conditioning for the competitive season began. Inclusion criteria were 14 y < age < 20 y, absence of major injuries in the preceding six months, absence of acute illness, and no medications affecting body composition or bone in the last year. Body mass was measured to the nearest 0.1 kg (Tanita electronic scale BWB-800 MA; Wunder SA.BI. Srl, Milano, Italy) and stature to the nearest millimeter using a Harpenden stadiometer (Holtain Ltd., Crymych, Pembrokeshire, UK), without shoes and with minimal clothing. Body mass index (BMI) was calculated as weight (kg) divided by height (m^2^). Players self-reported White or Black ethnicity as part of the demographic information collected during DXA acquisition; this information was recorded in the “Ethnicity” field of the Hologic DXA software where needed. All Black players immigrated in Italy from abroad within a maximum of four years. Since previous studies have reported differences in DXA-derived body composition across racial or ethnic groups [12,13,14], we used self-reported ethnicity in the analysis of body composition for the sake of comparison. Body composition (FM, %FM, and FFSTM) was evaluated using a total-body DXA scanner (QDR Horizon, Hologic Inc., Marlborough, MA, USA; fan-beam technology, software version 13.6.05). Quality control was performed daily using a manufacturer-supplied reference phantom to check for baseline drift. Hydration status was not objectively assessed in our sample. However, scans were performed in the late morning in a post-absorptive state and both the DXA lab and the waiting room were maintained at a constant temperature of 24 °C, thereby limiting changes in body water content. Further, participants were asked to refrain from vigorous exercise for at least 48 h before arriving at the laboratory. Measurements were performed according to Nana et al. (2015) [17]. Whole-body scans were taken. According to the convention of the International Society for Clinical Densitometry [18], in vivo short-term precision was calculated by repeated (n = 2) scanning of 30 subjects with repositioning; precision (percent coefficient of variation) was 2.12%, 0.59%, and 2.19%, for FM, FFSTM, and %FM, respectively. In whole-body scans, Hologic software divided the body into the trunk, both arms (left and right), and both legs (left and right), representing the TBLH and head regions. TBLH is recommended in younger subjects as the body composition outcome in adolescents [19]. However, in the Section 3, whole-body (TBLH plus head) values are also presented for comparison with previous literature. FM, %FM, and FFSTM were also calculated for the four limbs together (appendicular). One operator performed all scans and regional measurements to ensure consistency. Since stature is a confounding variable in a population of growing subjects, stature-adjusted indices such as the fat-free mass index (FFMI, FFSTM (kg)/stature^2^ (m)) and the fat mass index (FMI, FM (kg)/stature^2^) were also calculated.

To fully exploit the informing potential of chronological age in a sample of growing individuals, fractional age was calculated (YEARFRAC function in Microsoft Excel) and used throughout the analysis. In a growing population, maturity-related differences in both body mass and stature at the same chronological age [20] are found; accordingly, the maturity offset was estimated with the equation (R^2^ = 0.896, SEE = 0.542) proposed by Moore et al. (2015) [21] and used in the analysis.

### 2.3. Statistical Analysis

Data normality was assessed using the Shapiro–Wilk test. Data are presented as mean ± standard deviation (SD). Pearson’s correlation coefficient (r) and the partial correlation coefficient (r(PC)) were used to assess the bivariate and adjusted associations between variables, respectively. The strength of correlation was rated according to Hopkins [22]: small (0–0.30), moderate (0.31–0.49), large (0.50–0.69), very large (0.70–0.89), and almost perfect (0.90–0.99).

Percentiles for body mass, stature, and body composition variables were calculated by age group (14–15 y, 16–17 y, and 18–19 y). One-way ANCOVA in the general linear model was performed to evaluate differences in body composition across the four playing positions and to assess the effects of fractional age and ethnicity as covariates. The Levene test assessed homogeneity of variance, and the Breusch–Pagan test assessed heteroscedasticity. In case of significance, post hoc analysis with Bonferroni’s correction was conducted to evaluate differences between individual playing positions. Effect size (Cohen’s partial η^2^, η^2^_p_) was calculated and rated according to Cohen [23] as small (0.01), medium (0.06), and large (0.14).

The IBM SPSS Statistics (version 26.0; IBM Corp., Armonk, NY, USA) was used for all analyses. Statistical significance was set at *p* ≤ 0.05.

## 3. Results

### 3.1. Characteristics of the Study Sample

A complete dataset was available for 193 (goalkeeper, n = 19; defender, n = 60; midfielders, n = 76; forward, n = 38). Therefore, the study was sufficiently powered. In the whole sample, fractional age was 17.3 ± 1.06 y; body mass was 71.6 ± 6.97 kg; stature was 179.7 ± 6.14 cm; BMI was 22.1 ± 1.58 kg/m^2^. Eighty-nine percent of participants (n = 172) were White; 11% (n = 21) were Black, all of whom were outfield players.

Correlation analysis showed a small to moderate association between fractional age and body mass (r = 0.20, *p* = 0.006), BMI (r = 0.24, *p* < 0.001), TBLH FFSTM (r = 0.35, *p* < 0.001), appendicular FFSTM (r = 0.31, *p* < 0.001), TBLH FFMI (r = 0.42, *p* < 0.001), and appendicular FFMI (r = 0.39, *p* < 0.001). Body mass showed a very large correlation with stature (r = 0.70, *p* < 0.001), a small to large correlation with FM (r = 0.55, *p* < 0.001), TBLH %FM (r = 0.24, *p* = 0.001), TBLH FMI (r = 0.55, *p* < 0.001), and appendicular FMI (r = 0.51, *p* < 0.001). The correlation of body mass was almost perfect with TBLH FFSTM (r = 0.90, *p* < 0.001), very large with appendicular FFSTM (r = 0.85, *p* < 0.001), and large with TBLH FFMI (r = 0.55, *p* < 0.001) and appendicular FFMI (r = 0.51, *p* < 0.001). Stature showed a small to moderate correlation with TBLH FM (r = 0.40, *p* < 0.001) and TBLH%FM (r = 0.17, *p* = 0.018). Ethnicity showed a small correlation with TBLH %FM (r = 0.16 *p* = 0.029), appendicular FFSTM (r = 0.21, *p* = 0.004), TBLH FMI (r = 0.14, *p* = 0.050), and appendicular FFMI (r = 0.19, *p* = 0.009). After adjusting for fractional age, maturity offset, and ethnicity, the strength of the correlation between body mass and body composition variables slightly decreased, remaining statistically significant (*p* < 0.05); correlations between stature and body composition variables all became non-significant.

A one-way ANCOVA conducted across the whole sample, with fractional age as the factor and ethnicity and playing position as covariates, showed no statistically significant effect of either covariate on fractional age (ethnicity, *p* = 0.405; playing position, *p* = 0.173). When goalkeepers (n = 19) were compared with outfield players pooled together (n = 174), no statistically significant differences were found for body mass, stature, BMI, fractional age, and maturity offset (F from 0.028–0.971; *p* from 0.867–0.360); therefore, goalkeepers were included in subsequent analyses.

The body composition of White and Black players is reported in Table 1 and Table 2.

### 3.2. Comparison of White and Black Players

One-way ANOVA conducted in the whole sample with ethnicity as the factor showed that White players had lower whole-body and appendicular FFSTM with small to medium effect size (F = 4.112, *p* = 0.044, η^2^_p_ = 0.023 and F = 11.401, *p* = 0.001, η^2^_p_ = 0.062, respectively) as well as higher trunk FM and %FM with small effect size (F = 4.045, *p* = 0.046, η^2^_p_ = 0.023 and F = 5.200, *p* = 0.024, η^2^_p_ = 0.029, respectively). Similar results were found at the limit of statistical significance with a small effect size for TBLH FFSTM (F = 3.761, *p* = 0.054, η^2^_p_ = 0.021), and whole-body %FM and TBLH%FM (F = 3.746, *p* = 0.055, η^2^_p_ = 0.021 and F = 3.789, *p* = 0.053, η^2^_p_ = 0.022). Trunk FFSTM, whole-body FM, TBLH FM, appendicular FM, and appendicular %FM were not statistically significantly different in White and Black players.

As to stature-adjusted indices (Table 2), a statistically significant difference with a small effect size between White and Black players was found for appendicular FFMI (F = 6.992, *p* = 0.009, η^2^_p_ = 0.035), TBLH FMI (F = 3.870, *p* = 0.050, η^2^_p_ = 0.020), and trunk FMI (F = 7.145, *p* = 0.008, η^2^_p_ = 0.036). Whole-body FMI was borderline statistically significant with a small effect size (F = 3.593, *p* = 0.060, η^2^_p_ = 0.018). BMI, appendicular FMI, trunk FFMI, whole-body FFMI, and TBLH FFMI were not statistically significantly different with a small effect size. For all ANOVAs, the Levene statistic for homogeneity of variance was between 0.019 (*p* = 0.890) and 1.569 (*p* = 0.212) indicating that analysis was robust to the markedly different numerosity of White and Black players.

### 3.3. Playing Position and Body Composition

**Figure 1:**
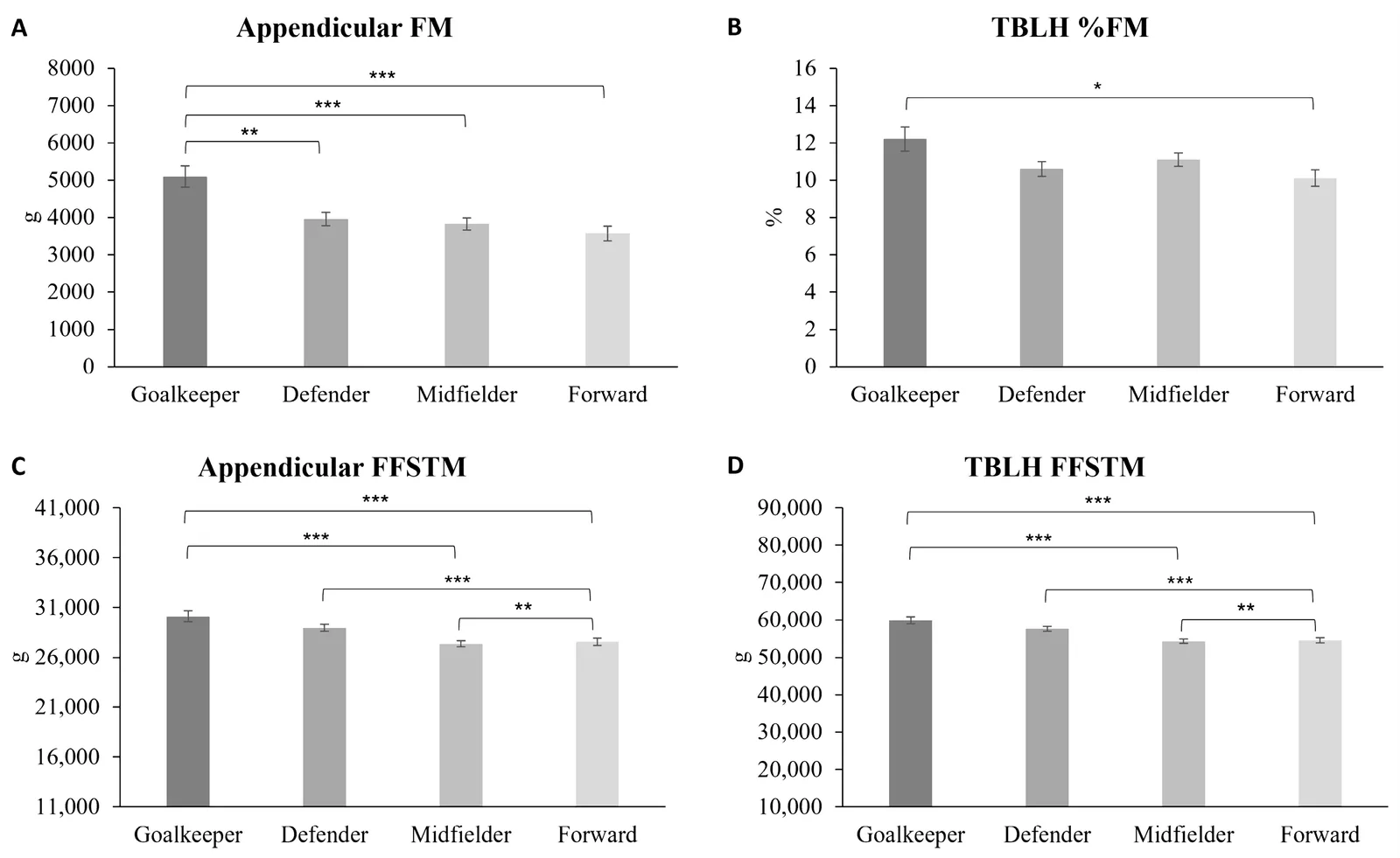
Panels (**A**–**D**). Comparison of selected body composition variables according to playing position (goalkeepers, defenders, midfielders, and forwards). Values are presented as estimated marginal means ± 95% confidence interval. FFSTM, fat-free soft tissue mass; %FM, percent fat mass; *, *p* < 0.05, **, *p* < 0.01, ***, *p* < 0.001).

In the position-specific analysis, one-way ANCOVA, conducted for body composition in the whole sample (n = 193) with playing position as the factor and fractional age, BMI, and ethnicity as covariates, showed a statistically significant effect of playing position with a medium to large effect size for whole-body, TBLH, and appendicular FM (*p* < 0.001 for all; F = 5.610–8.333, η^2^_p_ = 0.083–0.118). The effect of playing position on FFSTM was statistically significant at all sites (whole body, TBLH, appendicular, and trunk) (*p* < 0.001 for all; F = 12.791–16.134) with a large effect size (η^2^_p_ = 0.171–0.206). Percent FM was statistically significantly different across playing positions at all sites (*p* = 0.017–0.019; F = 3.388–3.494) with a small effect size (η^2^_p_ = 0.052–0.053). Post hoc analysis (Figure 1) showed that goalkeepers had higher FM than outfield players at the whole-body, TBLH, and appendicular sites (*p* = 0.004–< 0.001) as well as forwards at the trunk (*p* < 0.001). Goalkeepers showed a higher %FM than forwards at whole body (*p* = 0.021), TBLH (*p* = 0.016), and appendicular (*p* = 0.012), as well as defenders at appendicular (*p* = 0.041). Midfielders had a higher %FM in the trunk than forwards (*p* = 0.047). FFSTM was higher in goalkeepers than in midfielders and forwards at all sites (*p* < 0.001 for all). Defenders showed higher FFSTM than midfielders and forwards at whole body (*p* < 0.001, *p* = 0.001, respectively), TBLH, (*p* < 0.001, *p* = 0.001, respectively), appendicular (*p* = 0.001, *p* = 0.012, respectively), and trunk (*p* < 0.001, *p* = 0.001, respectively).

One-way ANCOVA, conducted for stature-adjusted body composition indices in the whole sample (n = 193) with playing position as the factor and fractional age and ethnicity as covariates, showed a statistically significant effect of playing position for FMI with a small to medium effect size at whole body, TBLH, and appendicular (*p* = 0.018–0.010, F = 3.431–4.993; η^2^_p_ = 0.052–0.073) but not at trunk (*p* = 0.066, F = 2.443; η^2^_p_ = 0.054). FFMI was statistically significantly different across playing positions with a small to medium effect size at all sites (*p* = 0.023–0.008, F = 3.243–4.026; η^2^_p_ = 0.049–0.061). Playing positions also differed for BMI with a small effect size (*p* = 0.015, F = 3.572, η^2^_p_ = 0.054). Post hoc analysis showed that goalkeepers had higher FMI than outfield positions at all sites except trunk (*p* = 0.031–0.002). FFMI was not significantly different across playing positions at WB (*p* > 0.05). At TBLH, appendicular and trunk FFMI were higher in forwards than midfielders (*p* = 0.009, *p* = 0.012, *p* = 0.030, respectively). No statistically significant difference in BMI was found across playing positions.

### 3.4. Percentiles

Percentile values of body composition variables for White players (n = 172) subdivided by age group (14–15 y, n = 23; 16–17 y, n = 101; 18–19 y, n = 48) are shown in Supplementary Table S1.

## 4. Discussion

### 4.1. Characteristics of the Study Sample

As expected in a growing population, fractional age, body mass, and stature were positively associated with body composition variables. Interestingly, goalkeepers were not statistically significantly different from outfield players in body mass, stature, and BMI, despite similar fractional age and maturity offset, indicating that the study group was sufficiently homogeneous in body size to be considered a single group in the analysis. The average whole-body %FM was comparable with that found in adult elite male soccer players (9.9–11.9%, [24]; 10.6%, [25]) and lower than in Premier League academy soccer players (around 14% in U16–U23 categories, [9]). Accordingly, the players in our sample demonstrated high fitness and could be considered representative of elite youth soccer players.

### 4.2. Comparison of White and Black Players

The role of ethnicity in influencing body composition in soccer players has been little investigated [25]. In this work, we showed (Table 1) that Black players have, compared with White players, higher mean lean mass and lower mean fat mass, a finding that was independent of stature at the appendicular, TBLH, and trunk sites. This is consistent with findings in the general population [12] and was not related to age differences, as mean FA was similar across the two player groups. Therefore, soccer participation does not appear to alter the body composition differences typically found between individuals of White and Black ethnicity. However, the observed differences should be interpreted cautiously due to the relatively low number of Black players in the sample and the lack of more detailed information on the players’ personal history.

### 4.3. Effect of Playing Position

Body composition, as well as anthropometry and physiological characteristics, are affected by playing position in soccer players [24]. After adjusting for confounding factors such as fractional age, ethnicity, and body size, we found that playing position affects FFSTM and absolute and relative FM at both the WB and regional levels. Similar results were obtained for stature-adjusted indices, indicating that stature is not a determinant of position-related differences in body composition. Post hoc analysis showed that goalkeepers differ in body composition from outfield players, especially for higher FM, consistent with a specific goalkeeper profile [24,25,26,27]. Here, we showed that FFSTM is also greater in goalkeepers (and defenders) than in midfielders and forwards, possibly in relation to larger body mass [15], whereas whole-body FFMI did not differ across playing positions. Interestingly, TBLH, appendicular, and trunk FFMI were higher in forwards than in midfielders, suggesting greater relative muscularity in the former. Although some position-related effects on FM and FFSTM were moderate to large, several effects for %FM and stature-adjusted indices were small to moderate. These findings suggest that positional differences may be relevant at the group level, while their practical importance for an individual player may vary. Accordingly, they should be interpreted alongside longitudinal within-player changes and other performance-related information when informing body-composition monitoring and training decisions.

### 4.4. Percentiles

Percentiles for demographic and body composition variables of youth elite soccer players, presented in Supplementary Table S1, were obtained for White players only, due to the limited number of Black players. Percentiles are useful in several sports codes, including soccer, for initial player assessment and monitoring [28,29,30,31]. Percentiles for anthropometric and body composition variables of adult male soccer players have been previously published ([32], n = 24; [28], n = 42), but these were obtained from a much smaller sample than the present study. In male youth soccer players, percentiles for DXA-measured body composition were obtained for 206 elite Brazilian youth players aged 11–16 years [10]. Data were presented for WB FM, FFSTM, and bone mineral mass according to maturity status. In this work, which involved a different, higher age range of participants (14–19 y), we also refrained from adjusting for maturity because all players showed a positive MO. Percentiles were calculated for FM and FFSTM at both the WB and regional levels, thereby providing more comprehensive information on the players’ body composition. The percentiles presented herein may provide descriptive data for youth soccer players but should not be considered general normative values.

### 4.5. Strength and Limitations

This work has limitations that should be acknowledged. First, players were recruited as a convenience sample from a single Serie A Club; therefore, the findings cannot be considered representative of the broader population of male youth soccer players. In particular, club-specific selection, training practices, and player characteristics may limit the external validity of the percentile values presented here; further, the number of Black players was limited, so comparisons with White players should be taken with caution. Second, we could not associate body composition and performance variables, thereby limiting the heuristic impact of the findings. Third, we did not investigate the possible association of dietary habits and body composition in the sample. Fourth, although DXA measurements were performed under standardized conditions broadly consistent with the best-practice recommendations for athletes proposed by Nana et al. [17], hydration status was not objectively assessed and may have introduced some variability in FFSTM estimates. Fifth, the cross-sectional design does not allow us to determine whether the observed body composition differences reflect player selection, maturation, or training-related adaptations over time. This work also has strengths that should be underlined: it was conducted in a large, a priori-determined sample of male elite youth soccer players, thereby ensuring adequate statistical power; it covered a large age span (14–19 years), providing sound data on body composition across adolescence; the use of DXA guaranteed accurate measurements of body composition variables; and descriptive percentiles were provided as a first reference for soccer participants and professionals.

## 5. Conclusions

This work characterized several fat- and lean-mass variables in a large sample of male elite youth soccer players overall and by playing position, thereby filling a gap in the literature. Moreover, percentiles for several demographic and body composition variables and indices were provided. These data may assist practitioners in benchmarking players and monitoring body composition development during adolescence. The limited effect of playing position suggests that body composition targets in elite youth soccer should be primarily individualized rather than position-specific.

## Figures and Tables

**Table 1:** DXA-measured body composition in White and Black male youth elite soccer players. Means ± SD.

Variable	Ethnicity	
	White (n = 172)	Black (n = 21)
WB FFSTM (g)	58,061.3 ± 5424.0 *	60,049.0 ± 6149.8
WB FM (g)	8344.2 ± 2097.2	7639.1 ± 1541.7
WB %FM	12.0 ± 2.4	10.8 ± 1.8
TBLH FFSTM (g)	54,945.2 ± 5433.1	56,795.2 ± 6110.0
TBLH FM (g)	7448.8 ± 2088.5	6698.4 ± 1570.4
TBLH %FM	11.4 ± 2.6	10.1 ± 2.0
Trunk FFSTM (g)	27,765.4 ± 2824.3	27,664.5 ± 2553.7
Trunk FM (g)	3420.9 ± 934.4 *	2944.9 ± 590.3
Trunk %FM	10.6 ± 2.3 *	9.3 ± 1.6
Appendicular FFSTM (g)	27,179.8 ± 2797.2 *	29,150.3 ± 3817.2
Appendicular FM (g)	4027.9 ± 1267.1	3753.5 ±1035.2
Appendicular %FM	12.2 ± 3.1	10.8 ± 2.6

DXA, dual-energy X-ray absorptiometry; WB, whole body; FFSTM, fat-free soft tissue mass; FM, fat mass; %FM, percent fat mass; TBLH, total body less head; *, *p* < 0.05 vs. Black.

**Table 2:** Stature-adjusted body composition indices in White and Black male youth elite soccer players. Means ± SD.

Variable	Ethnicity	
	White (n = 172)	Black (n = 21)
BMI (kg/m^2^)	22.1 ± 1.5	22.4 ± 1.6
WB FFMI (kg/m^2^)	18.00 ± 1.34	18.31 ± 1.43
WB FMI (kg/m^2^)	2.54 ± 0.57	2.32 ± 0.42
TBLH FFMI (kg/m^2^)	17.03 ± 1.32	17.31 ± 1.41
TBLH FMI (kg/m^2^)	2.30 ± 0.59 *	2.04 ± 0.43
Appendicular FFMI (kg/m^2^)	8.43 ± 0.71 *	8.88 ± 0.97
Appendicular FMI (kg/m^2^)	1.24 ± 0.36	1.14 ± 0.28
Trunk FFMI (kg/m^2^)	8.60 ± 0.67	8.43 ± 0.52
Trunk FMI (kg/m^2^)	1.06 ± 0.26 *	0.90 ± 0.16

BMI, body mass index; WB, whole body; TBLH, total body less head; FMI, fat mass index; FFMI, fat-free mass index; *, *p* < 0.05 vs. Black.

## Data Availability

The original contributions presented in this study are included in the article/Supplementary Materials. Further inquiries can be directed to the corresponding author.

## Supplementary Materials

The following supporting information can be downloaded at: https://www.mdpi.com/article/10.3390/jfmk11040417/s1, Table S1: Percentile (95% confidence interval, CI) values for White players’ body mass, stature, and fat and lean mass variables (n = 172).

## Abbreviations

The following abbreviations are used in this manuscript:

BMI	Body Mass Index
D	Defender
DXA	Dual-energy X-ray Absorptiometry
F	Forward
FM	Fat Mass
FMI	Fat Mass Index
FFMI	Fat-free Mass Index
FFSTM	Fat-free Soft Tissue Mass
GK	Goalkeeper
M	Midfielder
TBLH	Total Body Less Head
WB	Whole Body
