# Supplementary material for: Dual-Energy X-Ray Absorptiometry Investigation of Fat Mass and Lean Mass in 193 Male Elite Youth Soccer Players
Source: J Funct Morphol Kinesiol. 2026 Oct 13;11(4):417. doi: 10.3390/jfmk11040417 (PMC13692477; doi:10.3390/jfmk11040417)
Supplement: Supplementary file 1 [file jfmk-11-00417-s001.zip › jfmk-4474692-supplementary.pdf]

Table S1. Percentile (95% confidence interval, CI) values for White players' body mass, stature, and fat and lean mass variables (n = 172).

Variable	Age Group (y)	Percentile (CI)				
		10	25	50	75	90
Body mass (kg)	14-15	60.1 (55.4 – 63.9)	64.0 (60.5 – 66.7)	66.9 (64.8 – 70.2)	71.2 (67.6 – 74.5)	75.0 (71.3 – 84.4)
	16-17	63.6 (56.8 – 66.1)	67.2 (65.2 – 68.9)	71.0 (69.8 – 73.0)	77.6 (76.3 – 79.6)	82.3 (79.8 – 84.5)
	18-19	63.3 (56.8 – 66.1)	67.3 (65-6 – 70.2)	72.8 (69.2 – 76-4)	77.6 (76.3 – 79.6)	80.2 (78.4 – 81.5)
Stature (cm)	14-15	171.7 (158.1 – 173.7)	173.7 (171.8 – 176.8)	178.3 (174.4 – 180.9)	181.8 (178.9 – 184-4)	184.9 (181.7 – 185.8)
	16-17	173.7 (171.2 – 175.0)	176.4 (175.5 – 177.4)	179.2 (178.5 – 181.0)	184.5 (182.8 – 187.3)	189.9 (188.2 – 191.0)
	18-19	171.0 (168.1 – 173.3)	175.0 (172.7 – 176.1)	179.0 (176.3 – 180.3)	184.0 (180.3 – 189.0)	186.8 (185.3 – 187.8)
WB FM (g / 10^3))	14-15	6.4 (6.2 – 6.5)	6.5 (6.4 – 7.0)	7.2 (6.6 – 8.2)	8.4 (7.6 – 9.9)	10.3 (8.4 – 11.0)
	16-17	6.1 (55.5 – 6.6)	6.9 (6-7 – 7.1)	7.7 (7.5 – 8.5)	9.9 (8.9 – 10.3)	11.3 (10.5 – 12.4)
	18-19	5.7 (5.4 – 6.3)	7.1 (5.9 – 7.3)	7.9 (7.3 – 8.5)	9.0 (8.5 – 11.2)	11.5 (10.0 – 12.3)
WB FFSTM (g / 10^3)	14-15	48.1 (42.6 – 50.6)	50.4 (48.2 – 52.2)	53.1 (51.0 – 56.0)	57.9 (53.5 – 60.6)	61.3 (57.5 – 64.0)
	16-17	51.2 (50.2 – 53.3)	54.5 (53.5 – 55.5)	58.3 (57.1 – 57.9)	62.4 (60.1 – 67.5)	66.2 (64.2 – 67.5)
	18-19	53.7 (48.6 – 56.2)	56.8 (55.8 – 57.5)	60.3 (57.5– 62.3)	64.7 (62.2 – 66.0)	66.7 (65.5 – 68.3)
WB %FM	14-15	9.7 (9.0 – 10.7)	10.7 (9.9 – 10.9)	11.3 (10.8 – 12.4)	13.7 (11.8 – 15.0)	15.5 (13.7 – 16.8)
	16-17	9.2 (8.8 – 9.7)	10.2 (9.9 – 10.7)	11.5 (11.1 – 12.0)	13.1 (12.5 – 14.1)	15.3 (14.3 – 16.6)
	18-19	8.7 (7.8 – 9.5)	10.2 (9.3 – 10.5)	11.0 (10.6 – 11.6)	12.9 (11.6 – 14.8)	15.1 (13.1 – 16.0)
TBLH FM (g / 10^3))	14-15	5.4 (5.3 – 5.5)	5.6 (5.4 – 6.0)	6.2 (5.7 – 7.4)	7.6 (6.6 – 9.1)	9.4 (7.6 – 10.0)
	16-17	5.2 (4.7 – 5.7)	6.0 (5.7 – 6.2)	6.9 (6.6 – 7.5)	9.0 (8.1 – 9.5)	10.3 (9.6 –11.4)
	18-19	4.8 (4.5 – 5.3)	6.1 (4.9 – 6.4)	6.9 (6.4 – 7.6)	8.1 (7.6 – 10.3)	10.5 (8.9 – 11.5)
TBLH FFSTM (g / 10^3)	14-15	45.2 (3.7 – 47.0)	47.2 (45.3 – 49.3)	49.7 (48.0 – 52.7)	54.6 (50.4 – 57.2)	57.9 (54.7 – 61.2)
	16-17	48.2 (46.9 – 50.5)	51.1 (50.7 – 52.3)	52.2 (53.8 – 55.6)	59.1 (56.9 – 60.6)	63.1 (60.9 – 64.6)
	18-19	50.6 (45.8 – 53.0)	53.6 (52.5 – 54.4)	57.1 (54.4 – 58.8)	61.5 (58.9 – 62,1)	63.4 (61.9 – 64.9)
TBLH %FM	14-15	8.9 (8.2 – 10.0)	10.0 (9.2 – 10.3)	10.7 (10.0 – 12.5)	13.2 (11.2 – 14.6)	15.1 (13.3 – 16.5)

	16-17	8.4<br>(8.0 – 9.0)	9.6<br>(9.2 – 10.1)	10.9<br>(10.5 – 11.5)	12.6<br>(11.9 – 13.7)	15.0<br>(13.9 – 15.9)
	18-19	7.9<br>(7.2 – 8.7)	9.4<br>(8.5 – 9.9)	10.3<br>(9.8 – 11.1)	12.4<br>(11.0 – 14.1)	14.8<br>(13.6 – 15.8)
Trunk FM (g x 10^3))	14-15	2.3<br>(2.1 – 2.4)	2.4<br>(2.3 – 2.8)	2.8<br>(2.5 – 2.9)	3.0<br>(2.8 – 3.3)	3.6<br>(3.0 – 4.4)
	16-17	2.4<br>(2.3 – 2.6)	2.8<br>(2.6 – 2.9)	3.3<br>(3.0 – 3.4)	4.0<br>(3.7 – 4.2)	4.8<br>(4.4 – 5.3)
	18-19	2.3<br>(2.2 – 2.6)	2.8<br>(2.4 – 3.0)	3.3<br>(3.1 – 3.6)	3.7<br>(3.6 – 4.5)	4.7<br>(4.0 – 5.2)
Trunk FFSTM (g / 10^3))	14-15	22.4<br>(18.7 – 23.7)	23.7<br>(23.2 – 24.8)	25.2<br>(23.9 – 27.1)	27.6<br>(25.5 – 28.8)	29.1<br>(27.6 – 29.9)
	16-17	24.5<br>(23.9 – 25.1)	25.8<br>(25.3 – 26.2)	27.7<br>(27.2 – 28.4	29.9<br>(28.8 – 30.5)	31.3<br>(30.7 – 32.4)
	18-19	25.1<br>(23.6 – 26.5)	27.0<br>(25.6 – 28.0)	28.4<br>(27.9 – 29.6)	30.7<br>(29.5 – 31.7)	32.0<br>(31.4 – 32.3)
Trunk %FM	14-15	8.0<br>(7.6 – 8.8)	8.8<br>(8.4 – 9.3)	9.5<br>(8.8 – 10.2)	10.3<br>(9.7 – 11.6)	12.7<br>(10.3 – 15.1)
	16-17	8.1<br>(7.5 – 8.5)	9.0<br>(8.6 – 9.4)	10.3<br>(9.6 – 10.7)	11.8<br>(11.2 – 12.4)	14.2<br>(12.6 – 15.8)
	18-19	7.9<br>(7.3 – 8.2)	8.9<br>(8.1 – 9.7)	10.0<br>(9.6 – 10.5)	11.5<br>(10.4 – 12.6)	13.4<br>(11.8 – 14.7)
Appendicular FM (g / 10^3))	14-15	2.9<br>(2.6 – 3.2)	3.2<br>(3.0 – 3.3)	3.5<br>(3.2 – 4.5)	4.7<br>(3.8 – 5.2)	5.4<br>(4.7 – 6.4)
	16-17	2.6<br>(2.4 – 3.0)	3.1<br>(3.0 – 3.4)	3.7<br>(3.5 – 4.0)	4.9<br>(4.4 – 5.3)	5.6<br>(5.4 – 6.2)
	18-19	2.4<br>(2.2 – 2.6)	3.0<br>(2.5 – 3.5)	3.6<br>(3.5 – 4.0)	4.6<br>(4.1 – 5.2)	5.8<br>(4.8 – 6.9)
Appendicular FFSTM (g / 10^3)	14-15	22.4<br>(20.9 – 23.2)	23.6<br>(22.8 – 24.5)	24.7<br>(24.1 – 25.8)	26.3<br>(24.9 – 28.7)	29.7<br>(26.4 – 31.3)
	16-17	23.8<br>(22.9 – 24.7)	25.7<br>(24.8 – 26.2)	27.0<br>(26.5 – 27.5)	29.3<br>(28.1 – 30.3)	32.0<br>(30.4 – 33.0)
	18-19	24.7<br>(22.5 – 26.0)	26.4<br>(25.9 – 27.1)	28.2<br>(26.9 – 29.4)	30.5<br>(29.4 – 31.8)	32.2<br>(31.4 – 32.7)
Appendicular %FM	14-15	9.2<br>(8.6 – 10.6)	10.6<br>(9.7 – 11.5)	11.9<br>(10.7 – 14,5)	16-0<br>(12.2 – 17-4)	17.4<br>(15.9 – 17.9)
	16-17	8.5<br>(7.8 – 9,2)	10.2<br>(9.3 – 10.5)	11.7<br>(11.0 – 12.3)	13.8<br>(12.9 – 14.6)	15.3<br>(14.7 – 18.0)
	18-19	7.8<br>(7.4 – 9.1)	9.5<br>(7.9 – 10.2)	10.9<br>(10.2 – 11.8)	13.6<br>(11.7 – 14.6)	16.6<br>(13.9 – 17.2)

WB, whole body; TBLH, total body less head; FFSTM, fat-free soft tissue mass; FM, fat mass; FMI, fat mass index; FFMI, fat-free mass index.
